# Benign Osseous Metaplasia of the Breast: A Rare Breast Lesion—Case Report With Clinicopathologic Features and Review of the Literature

**DOI:** 10.1155/crip/8854614

**Published:** 2025-06-19

**Authors:** Mukund Tinguria, Angela Fleming

**Affiliations:** ^1^Department of Pathology and Laboratory Medicine, Brantford General Hospital, Brantford, Ontario, Canada; ^2^Department of Radiology, Brantford General Hospital, Brantford, Ontario, Canada

**Keywords:** benign osseous metaplasia, breast lump, breast needle core biopsy, breast ossification, mammography, nontumoral, ultrasound guided biopsy

## Abstract

Benign osseous metaplasia of the breast is an extremely rare breast lesion. This is a report of a 79-year-old woman who presented with a right breast lesion. The lesion was found incidentally on computed tomography (CT) scan examination of the chest. Subsequent mammogram showed coarse calcification within a round circular area measuring approximately 12 mm in size. Ultrasound examination showed an ill-defined 9 × 9 × 9 mm hypoechoic lesion with calcification and internal vascularity. Histologic examination of the excised lesion showed features of benign osseous metaplasia. There was no evidence of atypia and malignancy. The subsequent immunohistochemistry confirmed the diagnosis. The immunohistochemical staining for epithelial markers—pancytokeratin (AE1/AE3), Cam 5.2, HMWCK (34Be12), and CK5—was negative in the stromal component, which ruled out a metaplastic carcinoma. Osseous metaplasia occurs in association with a wide variety of benign and malignant breast lesions. However, primary benign osseous metaplasia in the absence of breast disease is an extremely rare entity. The case presented here is a reminder that osseous metaplasia can occur in the breast in isolation. The case also emphasizes the value of thorough histopathological examination in making the diagnosis, as clinical and imaging studies cannot differentiate between neoplastic and non-neoplastic lesions as well as benign and malignant neoplasms with certainty.

## 1. Introduction

Osseous metaplasia, also referred to as metaplastic ossification or heterotopic bone formation, can occur in a variety of neoplastic and non-neoplastic conditions at multiple body sites [1–5]. Osseous metaplasia in the breast is uncommon. The occurrence of bone and cartilage has been described in association with both benign and malignant breast neoplasms. Osseous metaplasia in the absence of breast disease is, however, exceedingly rare, with less than 10 cases being described in the medical literature [6–12]. The entity is diagnostically challenging, as it may mimic a neoplasm both clinically and on imaging studies. The aim of the report is to present clinicopathological features of primary nontumoral osseous metaplasia of the breast, emphasizing the importance of detailed pathological examination to arrive at a correct diagnosis. The report also describes differential diagnoses and pathogenesis theories of this rare entity.

## 2. Materials and Methods

### 2.1. Case Report

**Figure 1:**
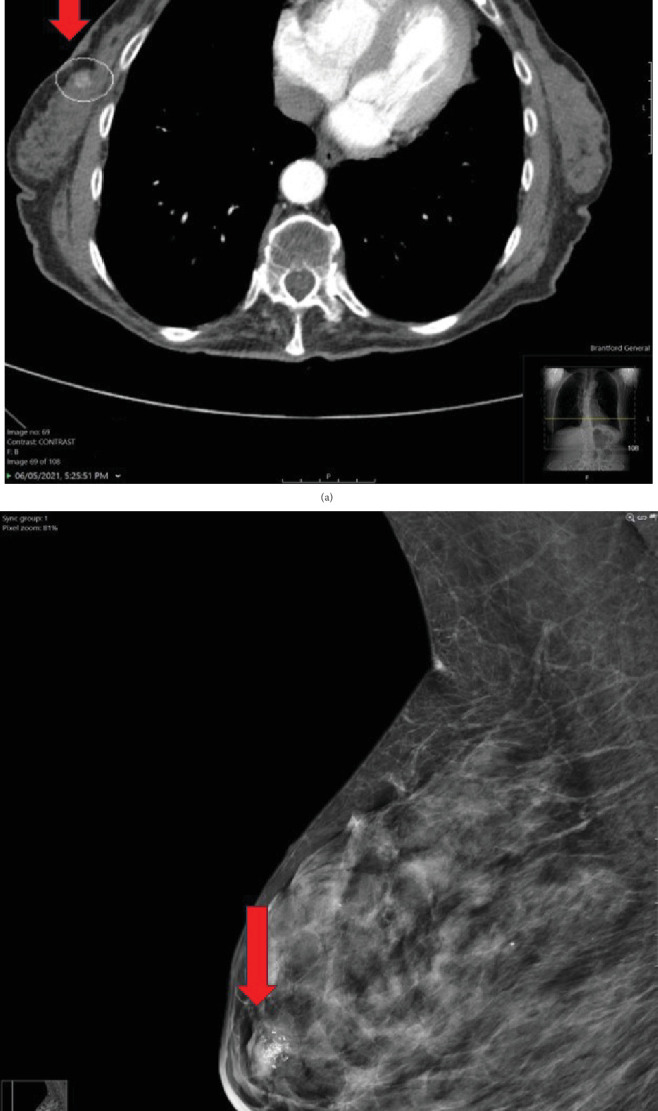
(a) Contrast-enhanced CT of the thorax performed to evaluate pulmonary nodule incidentally demonstrated a 1 cm nodular focus of increased attenuation within the right breast. (b) Subsequent mammogram demonstrated a partially calcified nodule in the right retroareolar region.

**Figure 2:**
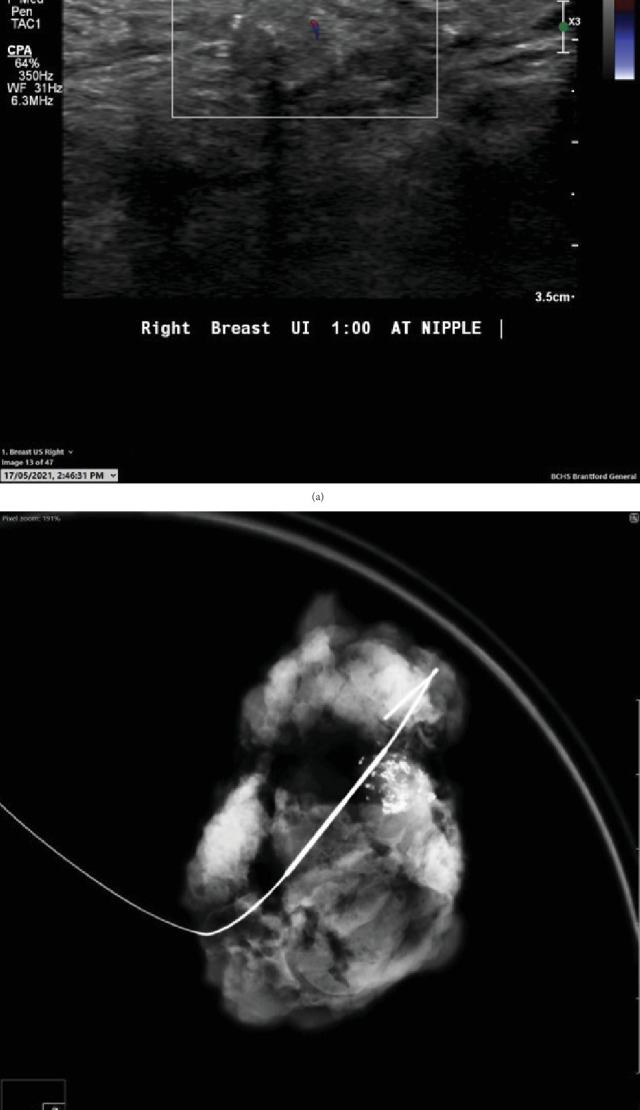
(a) Targeted right breast ultrasound demonstrated a 9 mm solid isoechoic to minimally hypoechoic nodule at 1 o'clock retroareolar region. (b) Specimen mammogram after needle localization and surgical biopsy. The localization wire was present in the specimen containing the partially calcified nodule.

A 79-year-old woman presented to the hospital with few months' history of weight loss and constipation. The colonoscopy revealed mild to moderate pancolonic diverticulosis and anal stenosis with tight anal sphincter. The esophagogastroduodenoscopy revealed a small hiatus hernia. There was no evidence of ulcer or a mass lesion in the gastrointestinal tract. To further investigate the weight loss, imaging procedure studies were carried out (see Table 1 for patient's diagnostic work-up). The CT scan examination did not show any tumor in the lungs, abdomen, and pelvis. A focus of increased attenuation was noted in the upper half of the right breast (Figure 1) and breast imaging procedure studies were recommended to further investigate this abnormal area. Subsequent breast mammogram showed coarse calcification within a round circular area measuring approximately 12 mm in size in the upper half of the right breast in subareolar region (Figure 1). This area conformed geographically and size-wise to the area of hyperdensity seen on CT scan of thorax. Review of previous imaging study documents showed that this area was present in the CT scan examination carried out 3 years before; however, it was smaller and less calcified that time. The differential diagnoses on mammogram included a calcified cyst. Ultrasound (US) examination of the right breast showed an ill-defined 9 × 9 × 9 mm isoechoic to minimally hypoechoic lesion with internal and peripheral calcifications and internal vascularity (Figure 2). In addition, mild ductal ectasia and a 5-mm cyst were noted at 9 o'clock radian. No other lesions were present. US examination diagnosis was that of a suspicious abnormality (BIRADS 4), and US-guided biopsy of the lesion was recommended for definitive pathologic diagnosis.

Imaging procedure studies of the left breast demonstrated a 1.5 cm nodule in the left axilla, which was present on numerous prior imaging studies. Imaging studies favored the diagnosis of a benign lesion such as a cyst or a lymph node.

### 2.2. Pathology

The needle core biopsy from the right breast lesion showed dense sclerosis with areas of osseous metaplasia. Mild inflammatory infiltrate consisting of lymphocytes, plasma cells, histiocytes, and foreign body type giant cells was present. Occasional benign normal breast ducts were noted. There was no evidence of atypia or malignancy. As osseous metaplasia can be associated with a wide variety of benign and malignant lesions, US-guided wire localization excision of the lesion was carried out for a definite pathologic diagnosis. The specimen mammogram after wire localization excision demonstrated a partially calcified nodule (Figure 2b).

### 2.3. Macroscopic Features

**Figure 3:**
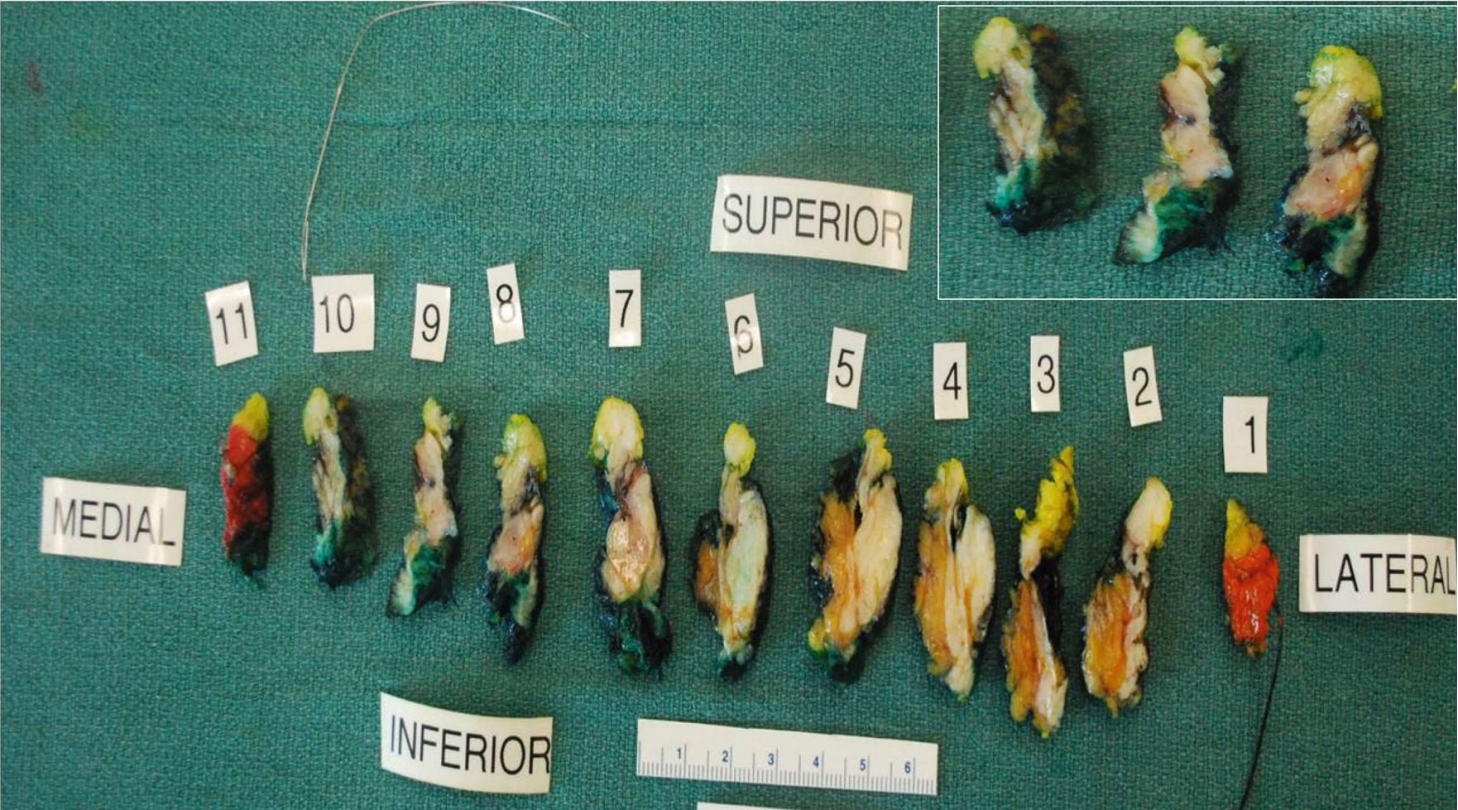
The specimen was sliced into 11 slices. Area of concern delineated with a localization was present in Slices # 8, 9, and 10 (see inset). This area did not look different from the surrounding breast parenchyma on gross examination, which showed ill-defined areas of fibrosis. As no tumor or an obvious lesion was identified on gross examination, the entire specimen was submitted for microscopic examination.

The specimen measured 6.2 × 4.7 × 1.8 cm. The area of concern was delineated with a localization wire. The specimen was sliced into 11 slices. Gross examination showed scattered ill-defined areas of fibrosis, but no obvious gross tumor was noted (Figure 3). The area of concern seen on the specimen mammogram did not look different from the surrounding breast parenchyma. As no tumor or an obvious lesion was identified on gross examination, the entire specimen was submitted for histologic examination.

### 2.4. Microscopic Features

**Figure 4:**
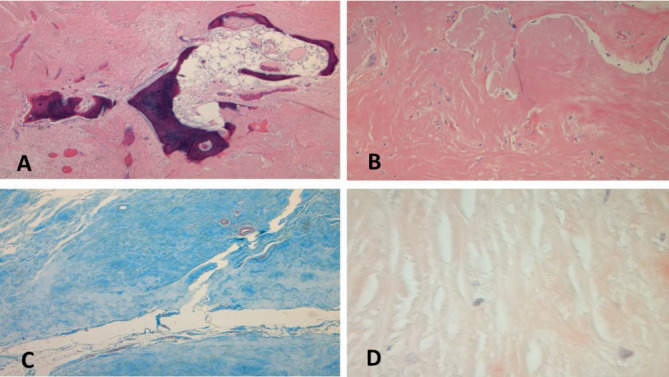
(A) Hematoxylin and eosin section from the lesion. An area of ossification was noted with mature lamellar bone surrounded by collagenous stroma (40X). (B) Surrounding fibrocollagenous stroma (100X). (C) Collagenous nature of stroma was further confirmed with Masson's trichrome stain which stains the stroma blue (100X). (D) Congo red stain: negative staining with Congo red stain ruled out amyloid deposits (100X).

**Figure 5:**
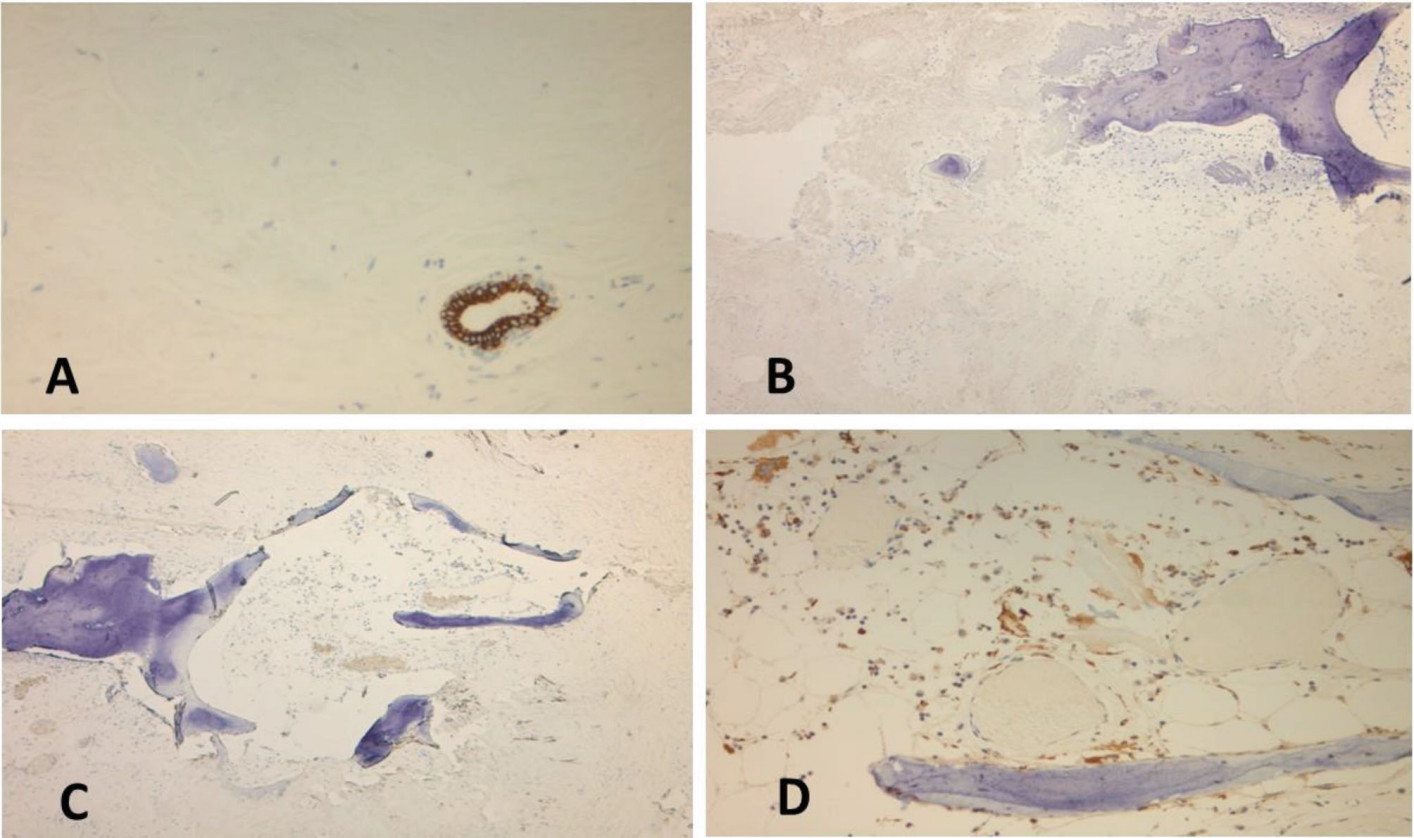
Immunohistochemical studies. The cytokeratin stains (pancytokeratin—AE1/AE3, Cam 5.2, and high molecular cytokeratin stain—34 BE 12) were negative in the stroma, which ruled out a metaplastic breast carcinoma. (A) Negative pancytokeratin (AE1/AE3) stain in the stroma. A benign duct showed staining with pancytokeratin stain (40X). (B) Negative staining with Cam 5.2 keratin marker (40X). (C) Negative high molecular weight cytokeratin (34BE12) stain in the stroma (40X). (D) CD68 positivity was noted in the histiocytic cells (40X).

Microscopic examination showed stromal sclerosis with dense paucicellular hyaline stroma and benign osseous metaplasia. Osseous metaplasia was characterized by mature bone fragments set in a fibrous stroma forming a mass-like lesion (Figure 4). Few scattered plasma cells, giant cells, and histiocytes were noted. There was no evidence of atypia and malignancy. The fibrous background was negative for amyloid on Congo red stain, which ruled out amyloid tumor (Figure 4). It showed features of collagen on trichrome staining (Figure 4). Epithelial markers pancytokeratin (AE1/AE3), Cam 5.2, HMWCK (34Be12), and CK5 were negative in the stromal component, which ruled out a metaplastic carcinoma (Figures 5, 5, 5, and 5). The surrounding breast showed features of fibrocystic change. The microscopic diagnosis was that of benign osseous metaplasia. Follow-up of the patient was recommended by regular breast imaging studies considering the rarity of the lesion with limited data available in the literature.

## 3. Discussion

Osseous metaplasia, also referred to as metaplastic ossification or heterotopic bone formation, can occur in a variety of neoplastic and non-neoplastic conditions at multiple body sites such as the endometrium, ovaries, thyroid, parathyroid, urinary bladder, thymus, scar tissue, and gastrointestinal tract, among others [1–5]. However, osseous metaplasia in the breast is rare. Bonet may have been the first to record a case of osseous metaplasia in the breast, describing a tumor that could not be cut with a knife [13]. Since then, there have been over 200 reports of osseous metaplasia in the breast associated with primary breast cancers, metastatic tumors of the breast, benign breast tumors, a variety of non-neoplastic conditions, and very rarely as an isolated finding.

Benign breast tumors showing focal osseous metaplasia include fibroadenoma, pleomorphic adenoma, benign mesenchymoma, phyllodes tumor, amyloid tumor, fat necrosis, atypical ductal hyperplasia, lipogranuloma, cholesterol granuloma, papilloma, hemangioma, breast abscess, chronic mastitis, fasciitis ossificans, and breast implant [14–30].

Malignant neoplasms showing osseous metaplasia include metaplastic breast carcinoma, invasive ductal carcinoma, fibrosarcoma, osteosarcoma, osteochondrosarcoma, malignant phyllodes tumor, and metastatic osteosarcoma to breast [19, 20, 31–38].

Osseous metaplasia in the breast in the absence of any other breast disease is extremely rare. Review of the literature revealed less than 10 cases of this rare entity [6–12]. The clinicopathologic features of benign osseous metaplasia of the breast are described in the table (Table 2). The age in reported cases ranged from 37 to 71 years. Most patients presented with a breast lump.

The imaging findings in primary osseous metaplasia of the breast are variable. In our case, mammogram demonstrated coarse calcification within a round circular area, and subsequent US showed an ill-defined hypoechoic lesion measuring 9 × 9 × 9 mm with internal and peripheral calcification and internal vascularity. The impression on imaging studies was of a suspicious lesion. Imaging findings in the reported cases of benign osseous metaplasia of the breast include suspicious looking lesions on mammogram and US examination [7], suspicious cluster of calcifications [6], cystic mass with areas of calcification [8], and features suggesting calcified fibroadenoma [9, 10].

Pathologic examination may or may not reveal gross lesions; when present, it is usually a well-circumscribed nodular lesion. Histologic examination shows densely sclerosed stroma with calcification and ossification with bone matrix, with osteocytes, osteoclasts, and spindle cells. Immunohistologic studies show negativity for epithelial markers such as pancytokeratin (AE1/AE3), Cam 5.2, HMWCK, MNF, CK5/6, p63, and CK14, which rule out metaplastic carcinoma [7, 12]. Pathologic examination is crucial for a definite diagnosis, as osseous metaplasia can be seen in a wide range of benign and malignant breast lesions. Benign osseous metaplasia, in the absence of other breast pathology, remains a diagnosis of exclusion when no other associated lesions are found on detailed pathological examination. The value of thorough sampling for pathologic examination cannot be emphasized to rule out foci of malignancy, considering prognostic and therapeutic implications.

Metaplastic breast carcinoma is one of the important differential diagnoses and needs to be ruled out by morphologic and appropriate immunohistologic studies. Metaplastic breast carcinoma represents a heterologous group of breast carcinomas characterized by differentiation of neoplastic epithelium towards squamous cells or mesenchymal appearing elements including but not limited to spindle, chondroid, and osseous cells. When these tumors lack a ductal carcinoma in situ (DCIS) or conventional mammary carcinoma component, a panel of immunohistochemical stains is required to confirm the presence of epithelial differentiation. Metaplastic carcinomas can be epithelial only carcinomas, pure (monophasic) sarcomatoid carcinomas, and biphasic epithelial and sarcomatoid carcinomas. Epithelial only carcinomas include low- and high-grade adenosquamous carcinomas and squamous cell carcinomas. Pure (monophasic) sarcomatoid carcinomas include fibromatosis-like metaplastic carcinoma and spindle cell carcinoma. Heterologous mesenchymal components include chondroid, osseous, rhabdomyosarcomatous, angiosarcomatous, and neurological differentiation singly or in combination. Mesenchymal components can show a wide spectrum of atypia ranging from minimal atypia to frank malignancy. The identification of the epithelial component may require extensive sampling and immunohistochemistry using broad spectrum keratin markers. Immunohistochemistry carried out in our case did not show positivity for epithelial markers pancytokeratin (AE1/AE3), Cam 5.2, CK5, HMWCK (34Be12), and p63, which ruled out metaplastic carcinoma. Smooth muscle myosin heavy chain (SMMHC-1) and p63 staining highlighted myoepithelial cells in benign ducts.

The pathogenesis of osseous metaplasia is yet to be completely understood. Suggested mechanisms include the transformation of stromal fibroblasts into osteoblasts, epithelial–stromal metaplasia, and ossification of calcific debris. The role of stem cells, cytokines, growth factors, extracellular matrix, and bone morphogenic proteins has been postulated [15]. In non-neoplastic lesions, ossification may be related to the underlying inflammatory processes such as osteoma cutis, which is related to chronic folliculitis [39]. Heterotopic bone develops when stromal fibroblasts are transformed by metaplasia into osteoblasts, and this metaplastic bone may arise directly from fibrous tissue or it can be secondary to cartilage formation [12]. It has been postulated that drug, metabolite, hormonal, and genetic factors also play a role in the development of osseous metaplasia [40].

## 4. Conclusion

Benign nontumoral osseous metaplasia of the breast is an extremely rare entity and may pose a diagnostic challenge clinically and on imaging studies. As clinical and imaging studies cannot differentiate between neoplastic and non-neoplastic lesions as well as benign and malignant neoplasms with certainty, histologic examination is necessary for definitive diagnosis. It is important for the pathologist to rule out other causes of osseous metaplasia in the breast before making a diagnosis of primary benign osseous metaplasia, which essentially remains a diagnosis of exclusion.

## Figures and Tables

**Table 1:** Patient's work-up of the right breast lesion.

**Procedure**	**Finding**
CT scan thorax to investigate patient's weight loss ↓	Showed a focus of increased attenuation in the right breast. This was an incidental finding. Breast imaging studies recommended.
Breast mammogram ↓	Showed coarse calcification in an area measuring approximately 12 mm. This area corresponded to the area seen on the CT scan of thorax.
Breast ultrasound ↓	Showed an ill-defined 9 mm lesion with calcifications. US examination diagnosis was that of a suspicious abnormality (BIRADS 4). US-guided biopsy recommended.
Ultrasound-guided breast biopsy ↓	Showed benign breast tissue with dense sclerosis and osseous metaplasia. Excision of the lesion recommended to rule out malignancy.
Wire localization excision of the breast	Gross examination showed areas of sclerosis. No obvious tumor was present on the gross examination. Microscopic examination showed dense sclerosis with hyalinized stroma and benign osseous metaplasia. There was no evidence of malignancy based on histology and immunohistochemical studies.

Abbreviation: BIRADS = Breast Imaging Reporting and Data System.

**Table 2:** Summary of the reported cases of benign nontumoral osseous metaplasia of breast.

**Reference**	**Age/sex**	**Presentation**	**Imaging studies**	**Pathology: Macroscopic features**	**Pathology: Microscopic features**
Gal-Gombos et al. [6]	37/F	Screening mammography	Suspicious cluster of calcifications on mammogram	Not described	Benign bone tissue in breast

Joshi et al. [7]	46/F	5 × 2 cm mass in left breast, with multiple lymph nodes in left axilla	Mammogram: 2.1 × 1.6 cm suspicious lobulated lesion Ultrasound: 2 × 1 cm lesion and axillary lymphadenopathy	Not described	Bone matrix with osteocytes, osteoclasts, and spindle cells Immunohistochemistry: Cytokeratin markers (AE1/AE3, MNF, Cam 5.2, CK5/6, CK14, and P63): Negative

Ansari et al. [9]	69/F	Left breast lump measuring 3 × 5 cm	Mammogram: Dense calcific lesion, likely calcified fibroadenoma Ultrasound: Suggestive of calcified fibroadenoma	Not described	Densely sclerosed stroma with calcification and ossification No evidence of malignancy

Alyami et al. [10]	38/F	1 × 2 cm mobile, firm nontender lump in the left breast	Mammogram: “Popcorn” calcifications, suggestive of calcified fibroadenoma Ultrasound: Suggestive of calcified fibroadenoma	Well-circumscribed whitish hard nodule.	Bone matrix deposition occupying most of the nodule with peripheral hyalinized tissue

Thushara et al. [8]	58/F	Left breast lump measuring 4 × 3 cm. No axillary lymph nodes	Ultrasound: Cystic mass with areas of calcifications	Cystic area measuring 3 × 2 cm	Sclerotic and elastotic stroma with foci of benign osseous metaplasia and calcifications. Adjacent breast showing dilated ductal–lobular units lined by metaplastic squamous epithelium

Ahmed et al. [12]	58/F	3 × 2 cm mass in the right breast	Mammogram: 2.7 × 2 cm lesion in the mid upper quadrant Ultrasound: Showed features suggestive of a calcified fibroadenoma	Bony hard tissue measuring 3.7 × 3.2 × 2.3 cm	Circumscribed mass composed of bone trabeculae and mature adipose tissue surrounded by hyalinized breast tissue Immunohistochemistry: Negative epithelial markers

Tinguria and Fleming, 2025 (current study)	71/F	Asymptomatic CT scan thorax to investigate weight loss showed a focus of increased attenuation in right breast leading to breast imaging studies	Mammogram: Coarse calcifications within a round circular area measuring 1.2 cm. Area corresponded to area of hyperattenuation Seen on CT scan. Ultrasound: Ill-defined hypoechoic lesion measuring 9 × 9 × 9 mm with internal and peripheral calcifications and internal vascularity Impression: Suspicious (BIRADS 4)	Ill-defined areas of fibrosis. No gross lesion	Stromal sclerosis with osseous metaplasia Immunohistochemistry: Epithelial markers (pancytokeratin [AE1/AE3], Cam 5.2, HMWCK [34Be12], and CK5)—Negative

Abbreviation: BIRADS = Breast Imaging Reporting and Data System.

## Data Availability

The data used to support the findings of this study are included within the article.

## References

[1] Bahceci M., Demirel L. C. (1996). Case Report: Osseous Metaplasia of the Endometrium: A Rare Cause of Infertility and Its Hysteroscopic Management. *Human Reproduction*.

[2] Singh P., Kapur K., Singla S., Naz N. (2011). Endometrial Osseous Metaplasia and Mature Bone Formation With Extramedullary Hematopoiesis. *Journal of Human Reproductive Sciences*.

[3] Chun S., Hong R., Jung A. (2013). Osseous Metaplasia With Mature Bone Formation of the Thyroid Gland: Three Case Reports. *Oncology Letters*.

[4] Byard R., Thomas M. (1988). Osseous Metaplasia Within Tumours. A Review of 11 Cases. *Annales de Pathologie*.

[5] Tica V., Postolache I., Boșoteanu M. (2023). Endometrial Osseous Metaplasia — A Rare Cause of Infertility With Unknown Etiology. *Medicina*.

[6] Gal-Gombos E., Esserman L., Poniecka A. (2002). Osseous Metaplasia of the Breast: Diagnosis With Stereotactic Core Biopsy. *Breast Journal*.

[7] Joshi M., Remoundos D. D., Ahmed F., Rees G., Cunnick G. (2013). An Unusual Breast Lump: Osseous Metaplasia. *Case Reports*.

[8] Thushara K., Rupashree S., Babu R. (2020). Benign Osseous Metaplasia of Breast – A Rare Case Report. *Annals of International Medical and Dental Research*.

[9] Ansari A. M., Dhillon K. S., Bhutani A. (2018). Benign Osseous Metaplasia: A Rare Breast Lump – Case Report. *International Journal of Scientific & Engineering Research*.

[10] Alyami H., al-Osail E., Harbi S., Bu Bshait M. (2018). Benign Osseous Metaplasia of the Breast: Case Report. *International Journal of Surgery Case Reports*.

[11] Steinberg J., D'Alfonso T., Eisen C., Arleo E. K. (2016). Osseous Metaplasia of the Breast Diagnosed From Stereotactic Core Biopsy: A Rare Entity With Radiologic-Pathologic Correlation. *Breast Journal*.

[12] Ahmed F., Abdelrahman S., Ibrahim A. (2020). Primary Osseous Metaplasia of Right Breast. *Open Journal of Pathology*.

[13] Bonet T. (1700). *Sepulchretum; Sive, Anatomía Practica, ex Cadaveribus Morbo Denatiis, Book 3, Chap. 01*.

[14] Fleming W. (1954). Cartilage and Bone Formation in Fibroadenomata of the Breast. *Australian and New Zealand Journal of Surgery*.

[15] Duhon D. J., Anton C. R., Ro J. Y., Venta L. A., Anton R. C., Schwartz M. R. (2021). Osseous Metaplasia in Hemangiomas of the Breast: Case Reports and Literature Review. *Journal of Breast Cancer*.

[16] Spagnolo D., Shilkin K. (1983). Breast Neoplasms Containing Bone and Cartilage. *Virchows Archiv. A, Pathological Anatomy and Histopathology*.

[17] Hota S., Giri R., Kabra H., Chauhan D., Pradhan P., Pujari P. S. (2021). Complex Fibroadenoma With Focal Ossification- An Enigmatic Finding in a Less Common Tumor. *International Surgery Journal*.

[18] Salh A., Abdullah A., Kakamad F., Hammood Z. D., Mikael T. M., Hassan S. H. (2022). Synchronous Occurrence of Breast Fibroadenoma With Osseous Metaplasia and Invasive Lobular Carcinoma; A Case Report With Literature Review. *International Journal of Surgery Open*.

[19] Smith B., Taylor H. (1969). The Occurrence of Bone and Cartilage in Mammary Tumors. *American Journal of Clinical Pathology*.

[20] Christensen L., Di Caterino T., Talman M. (2019). Phyllodes Tumor With a Benign Heterologous Osseous Component: A Diagnostic Challenge. *APMIS*.

[21] Sarkar S., Kapur N., Mukri H., Saurabh A., Kumar N. (2016). Chondroblastic Osteosarcoma of Breast in a Case of Phyllodes Tumour With Recurrence, A Rare Case Report. *International Journal of Surgery Case Reports*.

[22] Lynch L., Moriarty A. (1993). Localized Primary Amyloid Tumor Associated With Osseous Metaplasia Presenting as Bilateral Breast Masses: Cytologic and Radiologic Features. *Diagnostic Cytopathology*.

[23] Yokoo H., Nakazato Y. (1998). Primary Localized Amyloid Tumor of the Breast With Osseous Metaplasia. *Pathology International*.

[24] Lee H., Park S., Choi H., Park H. K. (2011). Lipogranuloma With Osseous Metaplasia in the Breast That Developed After “Bu-Hwang” Oriental Medicine Treatment. *Yonsei Medical Journal*.

[25] Garofalo S., Casolino C., Accurso A., Falleti J. (2008). Cholesterol Granuloma of the Breast With Unusual Ossification Features (Osseous Metaplasia). *Pathology, Research and Practice*.

[26] Imai S., Shima M., Sakamoto G., Ueda Y. (1984). A Case Report of Mastopathy With Cartilagenous and Osseous Metaplasia. *Gan no Rinsho*.

[27] Mannu G., Ahmed F., Cunnick G., Mungalsingh N. (2014). Bone Formation Within a Breast Abscess. *BML Case Reports*.

[28] Pohlodek K., Janík M., Mečiarová I., Ondriaš F. (2018). Pseudomalignant Myositis Ossificans in the Breast: A Case Report. *Molecular and Clinical Oncology*.

[29] Sato K., Oda Y., Ueda Y., Katsuda S. (2007). Fasciitis Ossificans of the Breast. *Pathology, Research and Practice*.

[30] Fan J. (2018). Rare Case of Osseous Metaplasia in the Setting of Saline Breast Implantation: A Case Report. *Clinical Radiology and Imaging Journal*.

[31] Yamaguchi R., Horii R., Maeda I. (2010). Clinicopathologic Study of 53 Metaplastic Breast Carcinomas: Their Elements and Prognostic Implications. *Human Pathology*.

[32] Lang R., Fan Y., Fu X., Fu L. (2011). Metaplastic Breast Carcinoma With Extensive Osseous Differentiation: A Report of Two Cases and Review of the Literature. *Tumori*.

[33] Reis-Filho J., Gobbi H., McCart R., WHO Classification of Tumours Editorial Board (2019). Metaplastic Carcinoma. *Breast Tumors*.

[34] Evans H., Shaughnessy E., Nikiforov E. (1999). Infiltrating Ductal Carcinoma of the Breast With Osseous Metaplasia: Imaging Findings With Pathologic Correlation. *American Journal of Roentgenology*.

[35] Gonzalez-Licea A., Yardley J., Hartman W. (1967). Malignant Tumor of the Breast With Bone Formation. *Studies by Light and Electron Microscopy*. *Cancer*.

[36] Jernstrom P., Lindberg A., Meland O. (1963). Osteogenic Sarcoma of the Mammary Gland. *American Journal of Clinical Pathology*.

[37] Kim J., Woo H., Kim E., Kim M. J., Moon H. J., Yoon J. H. (2016). Metastatic Osteosarcoma to the Breast Presenting as a Densely Calcified Mass on Mammography. *Journal of Breast Cancer*.

[38] Curran R., Dodge O. (1962). Sarcoma of Breast, With Particular Reference to Its Origin From Fibroadenoma. *Journal of Clinical Pathology*.

[39] Lui P., Pang L. M., Chu W. C. W., Tse G. M. K. (2004). Pathologic Quiz Case: A Solitary Breast Nodule in an Elderly Woman. *Archives of Pathology & Laboratory Medicine*.

[40] Kakamad F. H., Abdullah A. M., Salih A. M. (2021). Thymoma With Osseous Metaplasia; A Case Report With a Brief Literature Review. *International Journal of Surgery Case Reports*.

